# Case Report: Deep and durable PSA response to same-agent enzalutamide rechallenge in metastatic castration-resistant prostate cancer without chemotherapy

**DOI:** 10.3389/fonc.2026.1759642

**Published:** 2026-02-10

**Authors:** Huan Chen, Yan Wang, Min Qu, Xu Gao

**Affiliations:** The Department of Urology, Changhai Hospital, Naval Medical University, Shanghai, China

**Keywords:** androgen receptor signaling inhibitor, antiandrogen withdrawal, enzalutamide rechallenge, metastatic castration-resistant prostate cancer, treatment sequencing

## Abstract

Contemporary guidelines recommend docetaxel as first-line chemotherapy for chemotherapy-naïve men with metastatic castration-resistant prostate cancer (mCRPC) who progress on an androgen receptor signaling inhibitor (ARSI). Cabazitaxel is preferred after prior docetaxel and one ARSI. However, real-world constraints—such as patient preference, access, or comorbidity—often preclude chemotherapy, and cross-resistance among ARSIs is common, making robust responses to same-agent rechallenge uncommon. We report a 74-year-old man diagnosed in 2015 with *de novo* metastatic hormone-sensitive prostate cancer (Gleason 4 + 4; cT4N1M1b). Under continuous medical castration, he received sequential first- and next-generation ARSIs but consistently declined chemotherapy and radiotherapy. Following biochemical progression on enzalutamide in early 2025, which was accompanied by imaging findings suggestive of a new T12 lesion, and after a brief bicalutamide interval, enzalutamide was rechallenged under a time-limited, PCWG3-aligned protocol with predefined stop rules. Over five months, prostate-specific antigen (PSA) levels declined from 17.73 ng/mL to 2.62 ng/mL (~85% reduction); this PSA response was maintained for ≥5 months through the last follow-up while the patient remained on enzalutamide, without new adverse events or increased analgesic use. This case suggests that in a preference-constrained, chemotherapy-naïve patient, same-agent enzalutamide rechallenge can yield a clinically meaningful PSA response. The observation raises the hypothesis that ARSI resistance may be reversible in a subset of patients, warranting prospective evaluation of biomarker-guided rechallenge strategies to optimize patient selection and minimize opportunity cost.

## Introduction

Second-generation androgen receptor signaling inhibitors (ARSIs) such as enzalutamide and abiraterone have significantly prolonged patient survival and enhanced disease control in various stages of prostate cancer (1–3). Nevertheless, sequential ARSI exposure frequently induces shared resistance mechanisms that attenuate the efficacy of back-to-back ARSI therapy (4, 5). In men with metastatic castration-resistant prostate cancer (mCRPC), randomized trials have shown an overall survival benefit with docetaxel, and contemporary guidelines recommend docetaxel as the preferred first-line cytotoxic option for chemotherapy-naïve patients who progress on an ARSI (6–8). Switching to a second ARSI generally confers only modest benefit due to class cross-resistance (9).

Androgen receptor (AR) dependence may not be permanent. Under changing selective pressures, clonal composition and reversible epigenetic programs can shift over weeks to months (10–13). In patients in whom standard life-prolonging options are declined or not feasible, a short, prespecified re-exposure to the same ARSI may be considered as an exploratory probe of potentially reversible AR dependence, provided that explicit milestones and stopping rules are prospectively documented.

We report a decade-long mCRPC case illustrating that ARSI resistance is not necessarily absolute. Following progression on enzalutamide, a strictly time-limited rechallenge with the same agent—attempted after a brief off-enzalutamide interlude that included an ineffective bicalutamide course—produced a deep and durable prostate-specific antigen (PSA) decline in a patient who repeatedly declined chemotherapy and radiotherapy. This report details longitudinal PSA kinetics, evaluates alternative explanations for the response, and outlines a Prostate Cancer Working Group 3 (PCWG3)-aligned framework for exceptional use of same-agent rechallenge (14).

When patients decline chemotherapy and radiotherapy, clinicians must balance respect for patient preference with avoidance of therapeutic inertia. A short, predefined trial using the same ARSI, implemented with explicit milestones and stop rules, provides a cautious way to assess whether AR dependence can transiently return. Robust, durable biochemical responses to same-agent rechallenge after prior progression are uncommon in contemporary practice; most reports focus on switching to a different ARSI, which typically yields limited activity because of class cross-resistance (15–17). In the present case, the effect of reintroducing the same agent (enzalutamide) was isolated under continuous androgen-deprivation therapy (ADT) without additional systemic therapy, thereby probing whether previously observed resistance was reversible rather than fixed.

## Case report

Patient information. A 74-year-old man (body mass index 20.7 kg/m² at diagnosis) initially presented with severe lower urinary tract symptoms requiring palliative transurethral resection of the prostate. Available records provided limited comorbidity and family-history detail; no germline or somatic testing was performed because the patient declined molecular testing. Patient preference and financial constraints also limited access to advanced imaging (e.g., PSMA PET/CT) and serial biomarker assessments, and these factors were explicitly incorporated into shared decision-making.

### Initial presentation and staging

A 74-year-old man was diagnosed on 16 November 2015 with *de novo* metastatic hormone-sensitive prostate adenocarcinoma (Gleason 4 + 4 = 8). Baseline PSA was 600 ng/mL (23 November 2015). Whole-body MRI revealed tumor extension into the seminal vesicles, posterior bladder wall, and upper rectum; bone metastases in the bilateral inferior iliac bones, portions of the sacrum, and bilateral superior/inferior pubic rami; and multiple pelvic lymph node metastases, suggesting a clinical stage of cT4N1M1b. A baseline bone scan revealed no diffuse skeletal metastases. The patient underwent palliative transurethral resection of the prostate (TURP) and initiated continuous ADT with a GnRH agonist plus bicalutamide.

### Androgen-deprivation therapy phase (2015–2019)

Under combined ADT, PSA declined from 135.700 ng/mL in December 2015 to a nadir of 0.404 ng/mL in June 2019. Serum testosterone levels remained within the castrate range throughout.

### Transition to castration resistance and ARSI exposures (2019–2024)

By mid-2019, the disease had become castration-resistant, evidenced by a rising PSA reaching 0.639 ng/mL in May 2020. Adding flutamide was ineffective, with PSA increasing to 3.275 ng/mL by October 2020. Abiraterone plus prednisone (from November 2020) produced a temporary decline to ~0.58 ng/mL in July 2021, followed by a rise to 34.190 ng/mL by January 2023. Apalutamide was started in February 2023, lowering PSA to 2.666 ng/mL (May 2023), after which the PSA plateaued between 8–11 ng/mL over the subsequent year. Testosterone remained suppressed.

### First enzalutamide exposure and early progression (2024)

Enzalutamide began on 17 July 2024 (PSA 13.583 ng/mL). An initial decline to 8.798 ng/mL by 10 October 2024 was followed by biochemical progression, with PSA rising to 24.062 ng/mL on 2 January 2025. MRI on 14 February 2025 noted new findings (including possible involvement at T12) without visceral crisis. Denosumab, initiated in late February 2024 for bone health, was continued.

### Interlude and same-agent rechallenge (2025)

**Figure 1:**
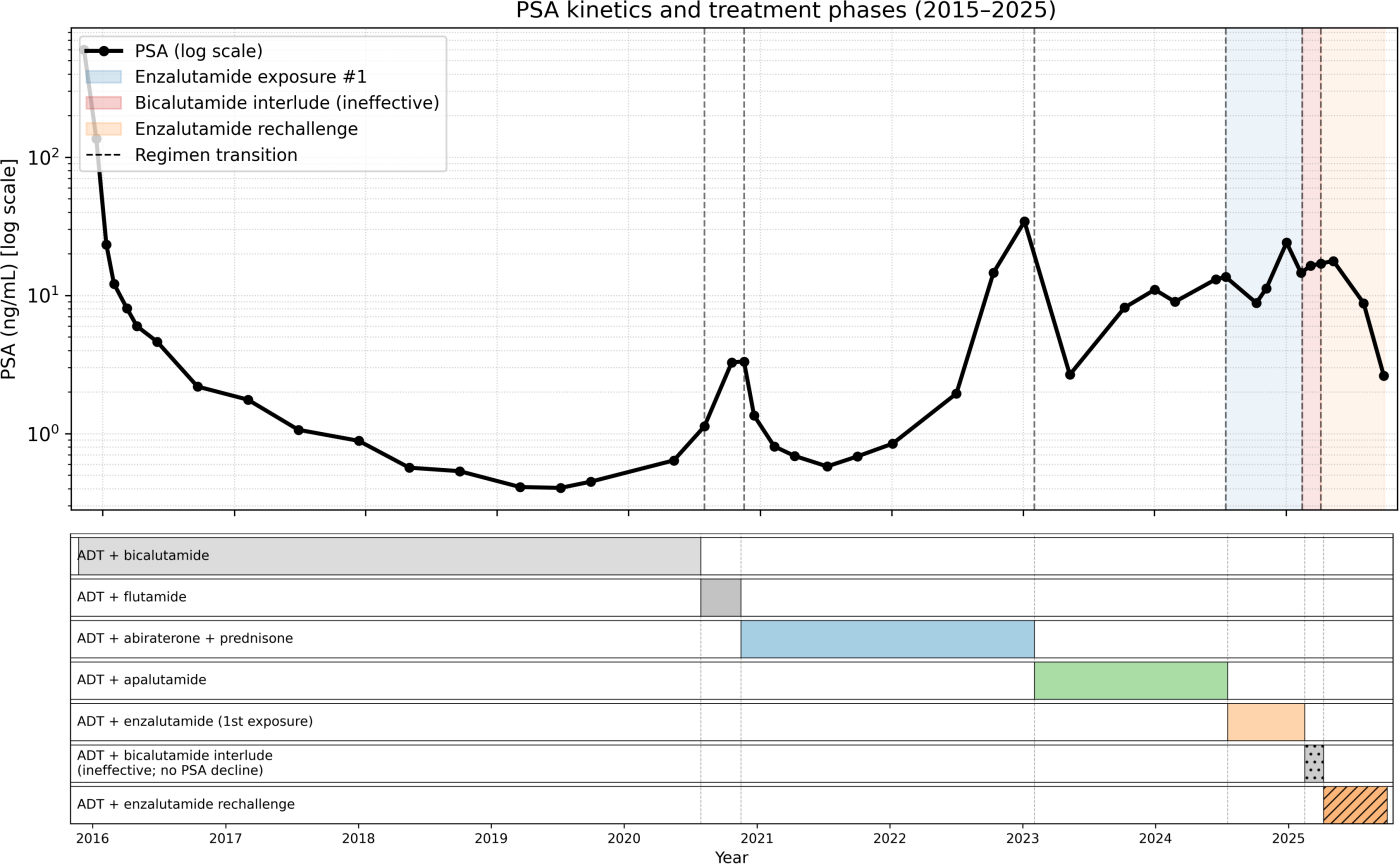
Longitudinal PSA kinetics and treatment timeline over a 10-year disease course. illustrates the longitudinal prostate-specific antigen (PSA) trajectory (upper panel) and corresponding systemic treatment phases (lower panel) over approximately 10 years in a patient with metastatic prostate cancer progressing to metastatic castration-resistant prostate cancer (mCRPC).

After confirming progression on enzalutamide, a brief bicalutamide interlude of approximately two months was undertaken in early 2025, during which PSA continued to rise (reaching 16.969 ng/mL by 7 April 2025). Enzalutamide was reintroduced on 7 April 2025 (with ADT maintained). PSA was 17.732 ng/mL on 12 May 2025 and then declined to 8.780 ng/mL on 4 August 2025 and 2.621 ng/mL on 29 September 2025. No other systemic anticancer therapy was administered during this interval (**Figure 1**).

### Clinical status, adherence, and safety

During rechallenge, the patient remained ambulatory with preserved daily function. There were no cancer-related pain crises or urinary obstruction events. Enzalutamide was administered at the standard dose with documented adherence; no dose reductions or interruptions were required. The patient reported mild fatigue without cognitive changes; blood pressure remained controlled. No falls or grade ≥3 adverse events occurred. Medication reviews confirmed the absence of strong CYP3A modulators, relevant antiepileptics, or chronic corticosteroids.

### Decision context

Despite counseling regarding evidence favoring docetaxel after ARSI failure, the patient declined chemotherapy and radiotherapy for personal and financial reasons. A PCWG3-aligned, time-limited enzalutamide rechallenge was therefore pursued with written documentation of biochemical and clinical stop criteria within a shared decision-making framework.

### Diagnostic and monitoring approach

Circulating tumor DNA (ctDNA) analysis for resistance biomarkers (e.g., AR-V7) was discussed but declined. Monitoring relied on serial PSA and clinical assessment; follow-up CT/MRI, bone scan, or PSMA PET/CT during the rechallenge interval was not obtained because the patient declined additional imaging, and radiographic reassessment was deferred unless symptoms evolved or biochemical control was not maintained.

### PSA confirmation

Each observed PSA decline was confirmed by a subsequent measurement ≥3 weeks later, consistent with PCWG3 recommendations (14), to minimize misclassification of random fluctuation as response. Symptom inventories and analgesic logs showed no worsening during PSA improvement.

At the time of reporting, the patient remained on enzalutamide, with the PSA response maintained for ≥5 months through the last follow-up (29 September 2025), which we define as durable in this report. Written informed consent for publication was obtained.

## Discussion

This case challenges the assumption that cross-resistance among ARSIs is uniformly absolute. In a preference-constrained setting, a pragmatic, time-limited approach was used to test whether AR dependence could re-emerge under continuous ADT. A same-agent enzalutamide rechallenge, implemented with prespecified milestones and strict stop rules and without confounding co-therapies, produced a deep and durable PSA response, indicating that resistance to an ARSI can be conditionally reversible in selected patients.

Limited literature suggests that same-agent enzalutamide rechallenge can occasionally regain activity. In a retrospective series (Sawazaki et al.), 2 of 8 patients (~25%) achieved a PSA50 response on enzalutamide rechallenge, supporting the concept that rechallenge may benefit select patients (18). Our patient’s ~85% PSA decline sustained over >5 months is larger than typically reported, highlighting that cross-resistance, although common, may not be absolute in every tumor state.

Several features support a causal relationship between rechallenge and PSA decline: appropriate temporality (decrease occurred only after reintroduction), internal consistency (serial, concordant declines), and a large effect size. Although PSA is an imperfect clinical surrogate, the magnitude and stability of reduction make random fluctuation an unlikely explanation.

According to contemporary guidelines, docetaxel is advised as the initial cytotoxic treatment for mCRPC patients who progress after a first ARSI and have not received prior chemotherapy. Furthermore, in men who have already undergone docetaxel after an ARSI, the CARD trial demonstrated that cabazitaxel outperforms a second ARSI, underscoring the cross-resistance observed with sequential ARSI use (19). In this case, the patient’s refusal of chemotherapy constrained options; rechallenge was therefore implemented as a short, well-guarded exploratory probe rather than a practice-changing strategy.

The quantitative pattern is informative. During the brief bicalutamide interlude (14 February–7 April 2025), PSA increased (16.371→16.969 ng/mL). Enzalutamide was reintroduced on 7 April; from the first post-rechallenge assessment (12 May, 17.732 ng/mL), PSA declined monotonically to 2.621 ng/mL by 29 September (~85% overall reduction). All measurements were obtained within the same laboratory network, and no clinical deterioration was observed, making chance variation, assay drift, or intercurrent prostatitis unlikely.

Other resensitization paradigms support biological plausibility. In PRESIDE, continuing enzalutamide while adding docetaxel modestly prolonged progression-free survival after initial enzalutamide resistance (20). Bipolar androgen therapy (BAT) has re-sensitized selected patients to subsequent enzalutamide (21), and a recent pooled analysis reported a PSA50 rate of 54% in 104 rechallenged patients (22). Although BAT was not used here, the durable decline with the same agent is consistent with a state-change model involving chromatin accessibility and co-regulator context.

The reversal of resistance in this context does not necessarily require fixed genetic changes (such as AR splice variants or ligand-binding domain mutations). Indeed, AR signaling outcomes depend on more than just genotype — they are influenced by the receptor’s ligand-induced conformational state, the availability of co-regulators, and interactions with other pathways (e.g., PI3K/AKT, MAPK), with the glucocorticoid receptor providing a potential bypass route. It is conceivable that purely epigenetic or phenotypic shifts over several weeks or months could switch an ARSI from acting like a partial agonist back to a full antagonist, thereby resensitizing the tumor cells in the absence of any new driver mutation (10, 23–25).

Patient selection is critical. Same-agent rechallenge is inappropriate in visceral crisis, rapidly progressive or highly symptomatic disease, or when patients are eligible for—and willing to receive—life-prolonging chemotherapy (8). Where robust resistance biomarkers (such as AR-V7 positivity) predict ARSI inactivity, effective alternatives should not be delayed (26).

Rigorous safety monitoring is essential during rechallenge. In practice, clinicians should proactively manage cardiovascular risk (e.g., frequent blood pressure checks and prompt treatment of hypertension) and mitigate fall risk (through patient evaluation and counseling). Patients should also receive guidance on maintaining good sleep habits and staying active to help counteract fatigue. Importantly, one should be ready to pause or reduce the enzalutamide dose at the earliest sign of significant (persistent grade 2–3) fatigue or hypertension (27, 28). Additionally, thorough medication review is needed to ensure the patient isn’t taking any strong CYP3A modulators or enzyme-inducing antiepileptic drugs that could alter enzalutamide exposure (29).

These observations support a dynamic model of AR signaling shaped by clonal competition, chromatin state, and pathway crosstalk (10). Given the modest and highly variable activity reported for ARSI cycling and the availability of standard life-prolonging options, same-agent rechallenge should not be routine. When evidence-based therapies are declined or unavailable, a strictly time-limited and well-documented rechallenge may serve as a low-cost biologic probe in carefully selected patients (30–32).

Ethical safeguards are required for exploratory rechallenges. Informed consent should specify the experimental nature of the approach, enumerate standard therapies with survival benefit that are being deferred, and delineate precise biochemical and clinical stop criteria. Multidisciplinary review is advisable when feasible.

Implementation should be prespecified. Before rechallenge, reconfirm castrate testosterone, review potential drug–drug interactions, and document baseline symptoms, performance status, and blood pressure. During rechallenge, measure PSA every 4–6 weeks with confirmation and discontinue if early milestones are not met (e.g., confirmed PSA50 by week 12–16 or at least arrest of further rise), unless there is compelling, patient-valued symptomatic benefit. Operational details are summarized in **Table 1**.

**Table 1:** Operational safety checklist for a time−boxed ARSI rechallenge.

Step	Action	Rationale	Timing
1	Confirm castrate testosterone (two values)	Avoid pseudo−progression from inadequate androgen suppression	Baseline; week 4
2	Reconcile DDIs (CYP3A modulators; antiepileptics)	Prevent altered exposure/toxicity	Baseline; each visit
3	Baseline PROs: pain/urinary; ECOG; BP	Anchor clinical benefit and safety thresholds	Baseline
4	PSA monitoring and confirmation	Detect early signal; avoid noise	Every 4–6 weeks; confirm PSA50 at ≥3–4 weeks
5	Pre−specified stop rules	Prevent therapeutic inertia	Biochemical ≥25% and ≥2 ng/mL above nadir; clinical/scan progression
6	Safety management (fatigue/BP/falls/cognition)	Mitigate ARSI toxicities	Each visit; home BP as needed
7	Biobanking/ctDNA (if feasible)	Enable translational analyses	Baseline; week 8–12; progression

Outcome assessment followed PCWG3 principles: the patient achieved a confirmed PSA50 response and continued therapy until PSA progression under prespecified stop rules, with clinical stability. Because transient PSA oscillations occur in a minority, confirmation is necessary to avoid misclassification (14).

Prospectively, a pragmatic registry could evaluate same-agent rechallenge within predefined guardrails. Serial CTC/ctDNA sampling (baseline, week 8–12, and at progression) could assess AR-V7 transcripts, AR ligand-binding-domain mutations and amplification, and exploratory glucocorticoid-receptor markers. Linking biomarker dynamics (e.g., AR-V7 clearance or declining mutant allele frequency) with PSA50 and PSA-PFS would inform biomarker-guided selection.

Generalizability is limited. The probability of benefit is expected to be low overall but non-zero in carefully selected patients, reflecting the balance of AR-driven versus AR-indifferent clones, epigenetic plasticity, and microenvironmental influences. The challenge is to identify this niche prospectively while minimizing opportunity cost.

Standardized documentation templates—capturing selection rationale, consent language, monitoring cadence, milestones, and exit criteria—can enable reproducible decisions and pooled analyses across centers, promoting equitable application.

Radiographic assessment warrants a staged approach. Imaging may lag biochemistry, and access to PSMA PET/CT is variable. For preference-constrained patients, anchoring initial decisions to biochemical criteria with confirmatory imaging after a sustained decline preserves timeliness without over-reliance on a single modality.

Potential confounders were assessed. Antiandrogen withdrawal is typically brief; here, the bicalutamide interlude lasted ~8 weeks (14 February–7 April 2025), during which PSA increased from 16.371 to 16.969 ng/mL, and there was no PSA decline after discontinuation (PSA 17.732 ng/mL on 12 May 2025) before subsequent decreases on enzalutamide rechallenge—arguing against this effect as the primary driver (33, 34). Denosumab is not known to induce substantial PSA reductions and was initiated in 2024, which is temporally inconsistent with the sustained 2025 decline. Analytical drift would not produce a smooth, months-long decrease. These considerations support a true pharmacologic effect of rechallenge.

Mechanistically, a renewed response to enzalutamide after prior progression can be conceptualized within a state-change framework rather than a single fixed genetic switch. Under changing selective pressure, resistant subclones may carry fitness costs, allowing relative re-expansion of AR-dependent, AR-V7–negative populations during an off-drug or low-pressure interval; in parallel, reversible chromatin and transcriptional programs can remodel AR cistromes and co-regulator usage over weeks to months. AR signaling output is also shaped by ligand-stabilized receptor conformation, co-regulator availability, and pathway crosstalk (e.g., PI3K/AKT, MAPK), with potential bypass via the glucocorticoid receptor. Together, these dynamics could restore net antagonist vulnerability without requiring a new dominant driver mutation. Importantly, because contemporaneous tissue or serial ctDNA/CTC profiling was not available, we cannot determine the relative contributions of AR-V7–negative clone re-expansion versus epigenetic reprogramming, nor can we track allele-frequency dynamics of AR ligand-binding-domain alterations; thus, the mechanistic interpretation remains hypothesis-generating (10, 23–25).

Biomarker-driven thresholds may aid decision-making. Achieving AR-V7 clearance in circulating tumor cells or a ≥50% reduction in the allele frequency of known AR resistance mutations by weeks 8–12 could support continuation; failure to meet biochemical or biomarker milestones should prompt discontinuation and transition to standard care or clinical trials. These thresholds are intended as hypothesis-generating and are extrapolated from emerging longitudinal AR-V7 and ctDNA data rather than prospectively validated cut-offs.

Bayesian adaptive trial designs may suit formal evaluation, given anticipated low response rates. Stratification by prior ARSI sequence, duration of initial ARSI benefit, and presence of visceral disease may improve efficiency while protecting non-responders (35, 36).

PROs should complement laboratory measures because biochemical responses do not always translate into symptom benefit. Brief validated instruments (pain scores, interference scales, urinary symptom indices) at baseline and each reassessment can keep decisions aligned with patient priorities; discordance should prompt re-evaluation of goals and consideration of alternatives with proven survival benefit (37, 38).

Limitations. As a single-patient observation, causal inference is limited and no conclusion regarding survival can be drawn. Follow-up cross-sectional or nuclear imaging after PSA decline was not obtained because of patient preference and access constraints (PSMA PET was unavailable), so contemporaneous radiographic confirmation of response is lacking. Most critically, without contemporaneous tumor tissue and serial ctDNA/CTC monitoring, we cannot distinguish re-expansion of AR-V7–negative clones from epigenetic reversal, quantify allele-frequency dynamics of AR alterations, or assess compensatory glucocorticoid receptor upregulation. Conclusions are therefore hypothesis-generating.

## Conclusion

In an mCRPC patient who declined chemotherapy, a direct same-agent enzalutamide rechallenge under continuous ADT produced a deep and durable biochemical response after prior progression on enzalutamide. Although cross-resistance among ARSIs is common, this single-patient case indicates that complete resistance is not inevitable and that ARSI sensitivity may occasionally re-emerge under defined conditions. A tightly time-limited, PCWG3-aligned rechallenge may be considered in exceptional, preference-constrained scenarios, but only with explicit stop rules, vigilant safety monitoring, and a low threshold for discontinuation if no benefit is observed. Prospective studies with standardized molecular correlates are required to determine whether a biomarker-defined subset might benefit from ARSI rechallenge; until such data exist, same-agent rechallenge should remain exceptional and protocolized.

## Data Availability

The original contributions presented in the study are included in the article/supplementary material. Further inquiries can be directed to the corresponding authors.
